# Lateral Compression Type 2 Pelvic Fractures—A Clinical Study of Fracture Displacement Measurement and Closed Reduction

**DOI:** 10.1111/os.13453

**Published:** 2022-08-31

**Authors:** Yan Wu, Hua Chen, Xuefeng Zhou, Peifu Tang

**Affiliations:** ^1^ Department of Orthopaedic Surgery General Hospital of Chinese People's Liberation Army Beijing People's Republic of China; ^2^ Department of Orthopaedic Surgery PLA Strategic Support Force Characteristic Medical Center Beijing People's Republic of China

**Keywords:** Closed reduction, Crescent fracture, Lateral compression injury, Pelvic fracture, Pelvic reduction frame

## Abstract

**Objective:**

To evaluate the displacement in four lateral compression type 2 (LC2) fracture subtypes (iliac wing and three kinds of crescent fractures) and to investigate the appropriate closed reduction for treatment using a pelvic reduction frame.

**Methods:**

A total of 71 patients with LC2 pelvic fractures from February 2014 to November 2019 were included in this retrospective cohort study. Preoperative X‐ray and computed tomography data were used to assess the direction of the fracture displacement and the sacroiliac joint dislocation. The fractures in all patients were reduced with a pelvic reduction frame and fixed with percutaneous screws as well as an anterior subcutaneous pelvic ring internal fixator. Two different closed reduction strategies were adopted, one was first longitudinal traction and then transverse traction, the other was first transverse traction then longitudinal and LC2 traction. The Matta score system was used to evaluate the postoperative X‐ray and the Majeed score system was used for follow‐up evaluation.

**Results:**

A total of 13 iliac wing fractures (86.7%) and 16 Day type 1 fractures (94.1%) were vertically stable with only internal displacement, the ring width displacements were 5 (3, 8.75) and 8 (4, 12) mm, the posterior superior iliac spine (PSIS) differences were 0 (0, 0) mm and 0 (0, 0) mm. A total of 21 Day type 2 fractures (95.5%) and 16 Day type 3 fractures (94.1%) were characterized by cephalic and dorsal fracture dislocation on the basis of internal displacement, the ring width displacements were 6 (4.25, 12) and 4 (0, 7.5) mm and the PSIS differences were 4 (2, 5) and 0 (0, 3.75) mm. Based on the Matta scores, excellent reduction was achieved in 51 patients, good reduction in 17 patients, and poor reduction in three patients. The average Majeed score was 91.6, with a minimum outpatient follow‐up of 12 months (average 31.6 months).

**Conclusion:**

LC2 fractures involve two different kinds of fracture displacement: internal displacement only and a combination of internal, cephalic, and dorsal dislocation through the sacroiliac joint. Good clinical outcomes can be achieved for LC2 fractures using two different closed reduction strategies.

## Graphical abstract

Abridged general view of the two different types of posterior ring injuries. Type1: iliac wing fracture and crescent fracture on Day 1 with the fracture line in a transverse direction and transverse displacement required longitudinal traction first (red arrow) followed by transverse traction (blue arrow). Type 2: On Day 2 and Day 3 with fracture line in a longitudinal direction and dislocation as well as longitudinal displacement required transverse traction first (red arrow) followed by longitudinal and LC2 traction (blue arrow).

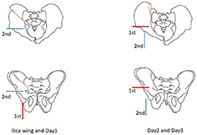

## Introduction

Lateral compression (LC) pelvic injuries account for nearly 80% of all pelvic ring injuries^1^, ^2^, ^3^, ^4^ and are often described as “implosions” caused by a violent inward force. According to the Young–Burgess classification system for LC injuries, LC pelvic fractures can be classified into three types (LC1, 2, and 3).^5^ Under the classification system introduced by Day *et al*., LC2 pelvic fractures are a complicated subset of fractures with four distinct subtypes.^6^, ^7^ One is the iliac wing fracture, in which the fracture line is located between the sacroiliac and hip joints. The other three are crescent fractures that involve fracture dislocation of the iliac wing through the sacroiliac joint and can be divided into three distinct subtypes according to the extent of sacroiliac joint involvement and the size of the crescent fragments.^7^

The current standard treatment for LC2 fractures is open reduction and internal fixation. Both methods require muscle detachment from a large area, which causes substantial damage.^2^, ^3^, ^7^ Therefore, closed reduction for pelvic fractures holds substantial promise, and many pelvic closed reduction tools have been invented,^8^, ^9^, ^10^, ^11^ such as the Matta pelvic frame and Starr frame, which can provide intraoperative transverse traction.

Regardless of the closed reduction tools used, understanding the rule of fracture displacement is a prerequisite for anatomical reduction. However, due to the complexity of LC2 fracture classification, the number of fractures categorized as each of the four subtypes is relatively small. Systematic descriptions of fracture morphology and reduction mechanisms are also lacking.

The aims of this study were (i) to evaluate the displacement in LC2 fractures and (ii) to investigate the appropriate closed reduction strategy for treatment using a pelvic reduction frame. We retrospectively evaluated 71 cases of LC2 fracture treated with closed reduction techniques using an external pelvic reduction frame and summarized the closed reduction strategies for LC2 fractures.

## Patients and Methods

### General Data

This retrospective cohort study was completed at a level I trauma center following approval by the medical ethics committee of PLA General Hospital (No.2019092656455).

The inclusion criteria were: (i) LC2 pelvic fracture (Orthopaedic Trauma Association classification [OTA] B2.2); (ii) age ≥15 years old; (iii) surgical treatment was performed with closed reduction and internal fixation. The fracture classification was determined by the attending orthopaedic trauma surgeon. The decision for treatment was at the discretion of the attending orthopaedic trauma surgeon with input from the patient and/or their family. The exclusion criteria were: (i) pathological fractures; (ii) severe medical complications or other associated severe injuries, such as nerve injury or vascular injury.

From February 2014 to November 2019, 75 patients with LC2 pelvic injuries were treated with closed reduction and internal fixation, and 71 of these patients with a minimum outpatient follow‐up of 12 months were included in this study. Four patients were excluded from the study because they were lost to follow‐up. The demographic patient data, surgical details, and patient history were obtained from medical records and a spreadsheet was used for data entry (Table 1).

**TABLE 1:** Patient characteristics

Characteristics	All patients	Iliac wing	Day type 1	Day type 2	Day type 3	*χ* ^2^ or *F* value	*p* value
Age (years)	52.0 ± 19.1	58.4 ± 22.9	52.1 ± 21.7	49.4 ± 15.1	51.1 ± 16.0	1.283	0.2877
Sex (M:F)	31:40	6:9	8:9	12:10	5:12	0.6257	0.5739
BMI (body mass index, kg/m^2^)	23.4 ± 3.6	24.1 ± 4.6	22.6 ± 2.3	23.6 ± 3.3	23.4 ± 4.1	0.6645	0.5770
Injury mechanism						4.296	0.0386
Traffic accident	31	4	5	12	10		
High fall	24	6	8	6	4		
Crush	6	0	0	3	3		
Ground fall	10	5	4	1	0		
Time to surgery (days)	11.4 ± 6.1	9.9 ± 4.5	12.0 ± 6.6	11.1 ± 6.5	11.4 ± 6.9	0.5881	0.4918

According to the classification systems described by Young–Burgess and Day *et al*.,^6^, ^7^ the 71 patients were classified into four groups. A total of 15 patients were in the iliac wing group and 56 patients with crescent fractures were classified as follows: 17 in the Day type 1 group, 22 in the Day type 2 group, and 17 in the Day type 3 group.

### Imaging Examination and Displacement Measures

All patients received X‐ray (anteroposterior [AP], inlet, and outlet views) and computer tomography (CT) examinations before the procedure. Radiographs and CT images were available for review from the picture archiving system and the fracture and dislocation patterns were analyzed using coronal and transverse sections in the preoperative CT images. Based on the CT results, the fractures and dislocation could be defined as longitudinal or transverse. The greatest displacement on the injured side was measured and compared to the healthy side as a standard reference.^12^ The displacement of the iliac wing height, sacral height, and ischial height was measured with the AP view. The displacement of the PSIS, sacral width, and ring width was measured with the inlet view, and the displacement of the iliac wing height and ischial height was measured with the outlet view.^11^, ^12^

### Surgical Process

Step 1: The operation was performed in the supine position on a radiolucent surgical table. A 3–4 cm thick cushion was placed under the sacral region.

**FIGURE 1:**
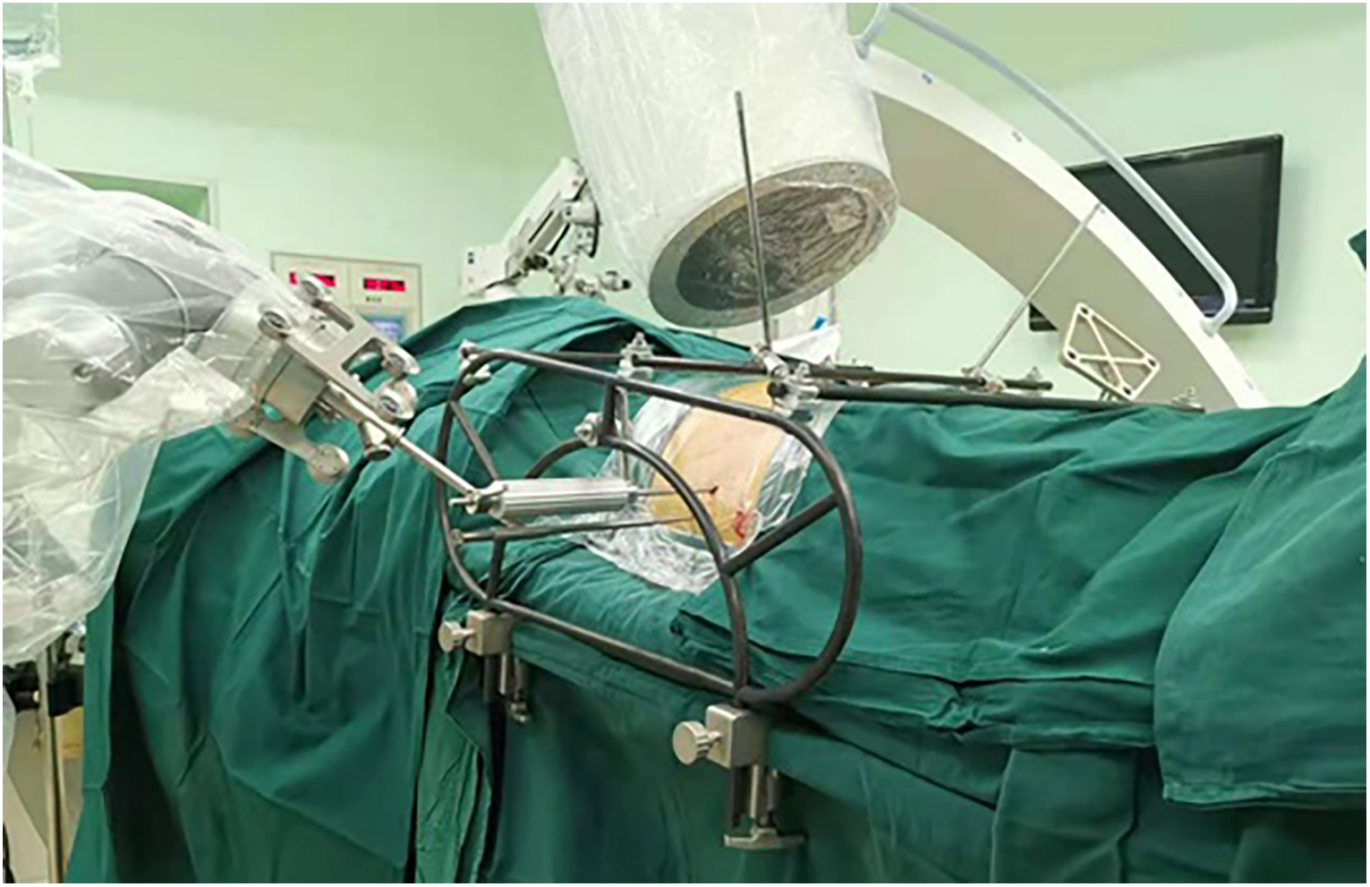
Pelvic reduction frame cooperation with the C‐arm and the navigation robot

Step 2: The femoral supracondylar bone traction was connected to the traction bed, but no perineal post was used after conventional disinfection was completed. Afterward, a pelvic closed reduction frame (Beijing Guoxietang Technology Development Co., Ltd) was installed for assistance (Figure 1). In order to stabilize the healthy side as the reduction reference, two long Schanz screws were inserted into the uninjured pelvic ring and connected to the frame, one at the lateral–medial supra‐acetabulum and the other through the LC2 channel. To control the reduction, two more long Schanz screws were inserted on the injured side in the same manner.

**FIGURE 2:**
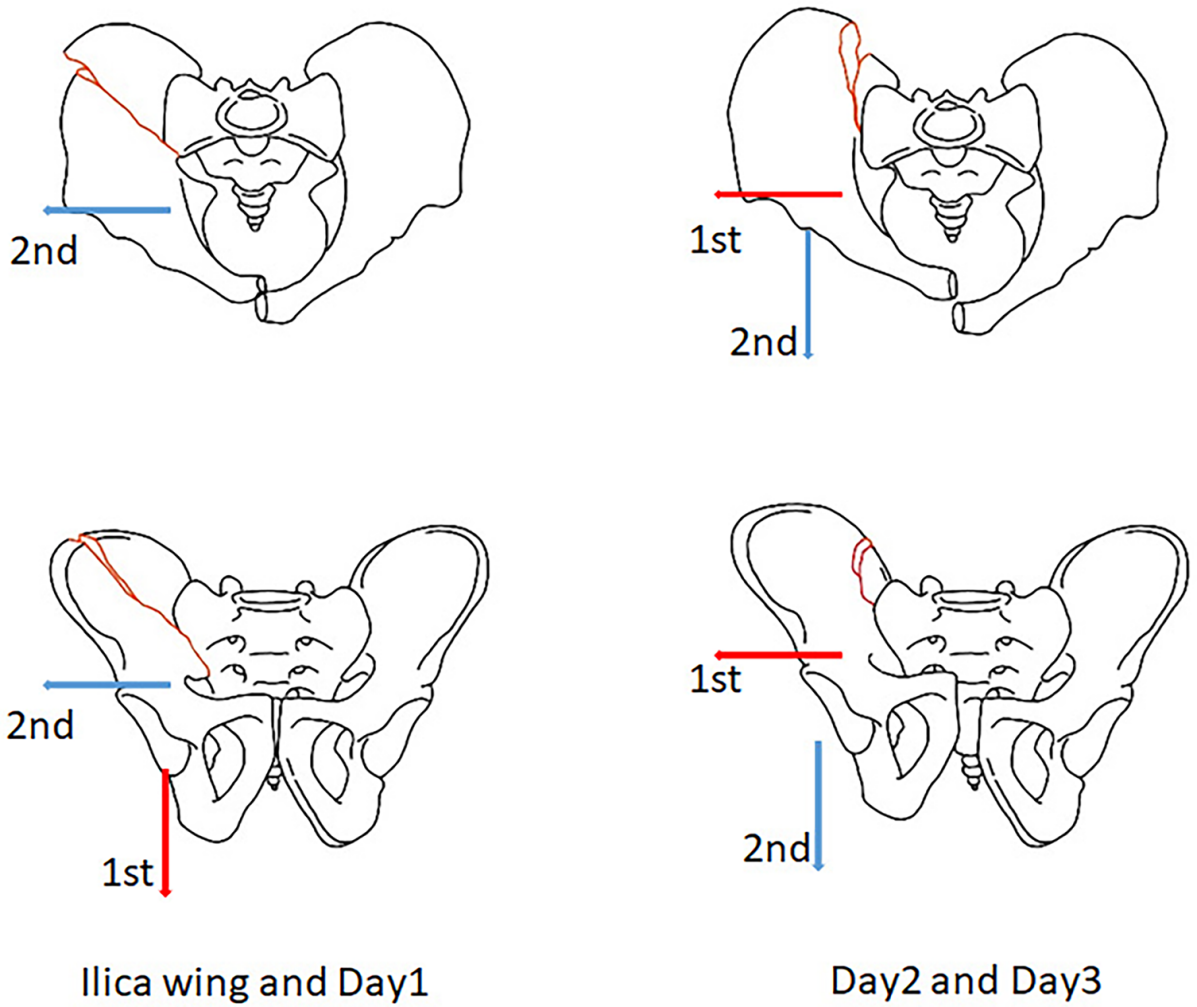
Abridged general view. Type 1: iliac wing fracture and Day type 1 crescent fracture with the fracture line in a transverse direction and transverse displacement required longitudinal traction first (red arrow) followed by transverse traction (blue arrow). Type 2: Day types 2 and 3 crescent fracture with fracture line in a longitudinal direction and dislocation as well as longitudinal displacement required transverse traction first (red arrow) followed by longitudinal and lateral compression type 2 (LC2) traction (blue arrow)

Step 3: After the above preparations were completed, the reduction of the fracture began.^13^ Abridged general view showed the two different closed reduction strategies for two different types of posterior ring injuries (Figure 2).

For iliac wing and Day type 1 fractures, it is necessary to reduce the misplacement of the median sagittal section and transverse section for easier observation of the reduction using fluoroscopy. This can be achieved by adjusting the two long Schanz screws inserted on the injured side simultaneously. Femoral supracondylar bone traction was first performed to separate the impacted portion of the iliac wing, and the iliac wing height and ischial height were used to evaluate the reduction. Slight over‐traction can be helpful for the reduction during this process. The width of the pelvic ring was next reduced using the lateral–medial supra‐acetabulum screw traction. The femoral supracondylar bone traction was released when the ring width was deemed acceptable in the inlet view. Afterward, slight adjustments were made to the two long Schanz screws to fine tune the misplacement of the median sagittal section and transverse section.

For the Day types 2 and 3 crescent fracture dislocation groups, the rotational displacement was first reduced. Afterward, traction of the lateral–medial supra‐acetabulum screw was used to separate the impacted portion of the posterior sacroiliac joint. The femoral supracondylar bone traction was used to reduce the iliac wing height and ischial height. The displacement of the PSIS difference was reduced through the traction of the LC2 screw. The lateral–medial supra‐acetabulum screw was then used to correct the sacroiliac joint dislocation. Lastly, the two long Schanz screws were adjusted to fine‐tune the misplacement of the median sagittal section and transverse section again.

**FIGURE 3:**
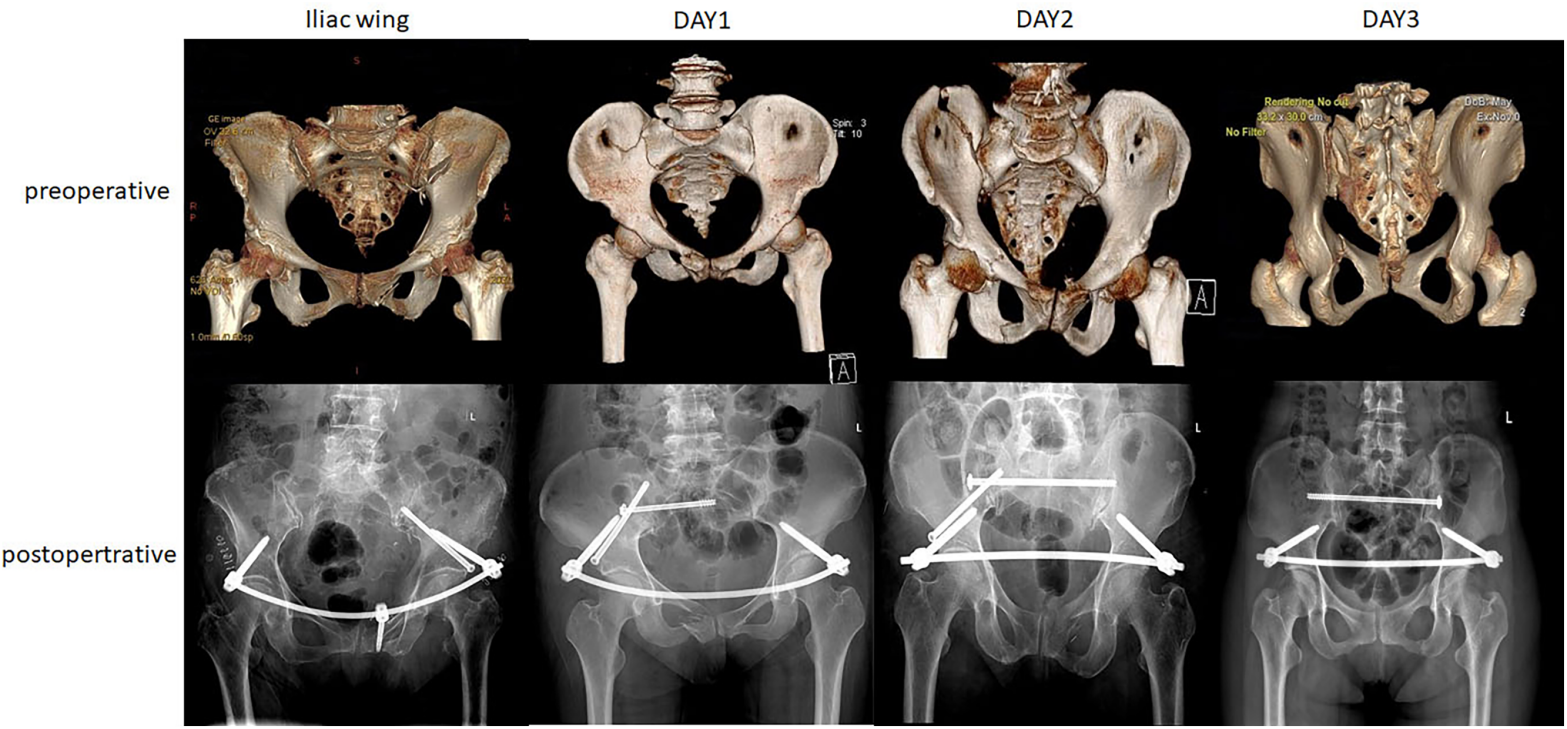
Typical cases from each group with preoperative and postoperative images

Step 4: When all the reduction indexes were satisfactory, posterior fracture fixation was performed with percutaneous screws (7.3 mm partially threaded screw). LC2 screws were used to treat iliac wing, Day type 1, and Day type 2 fractures. Sacroiliac joint screws were used to treat some crescent fractures, mostly Day types 2 and 3 fractures.^6^, ^7^ Anterior ring fractures were fixed with an anterior subcutaneous pelvic ring internal fixator (Figure 3). The pedicle screws were inserted into the channels drilled along the long LC2 Schanz screws once the external fixator frame was withdrawn. Finally, the position of the pelvic ring was confirmed with fluoroscopy. In 26 cases, percutaneous screws were planned by an orthopaedic robot (TINAVI Medical Technologies Co. Ltd., China), and the screws in the other 45 cases were inserted with fluoroscopy guidance.

### Matta Radiological Scoring and Majeed Functional Scoring

Matta standard results were used to evaluate the fracture reduction based on radiographs and CT images obtained postoperatively. Fracture reduction was rated as excellent (<5 mm), good (5–10 mm), fair (11–20 mm), and poor (>20 mm).^14^ The Majeed score system was used for functional assessment during the follow‐up period.^15^ Follow‐ups were conducted at 1, 3, and 6 months after the operation and every half year thereafter.

## Statistical Analysis

SPSS statistical software (version 26.0, International Business Machines, Corp.) was used for statistical analysis and the data were used to generate the median (interquartile range) values for the groups because the displacement did not follow a normal distribution due to the classification system. Analysis of variance and chi‐square tests were used to analyze the correlations between the four groups. Significant level *α* = 0.05.

## Results

### Pattern of Fracture and Dislocation

#### Fracture Line Analysis

According to the preoperative CT examination, among 15 cases in the iliac wing group, 13 cases involved a fracture line in the transverse direction and two cases involved the longitudinal direction. In the Day type 1 group, there were 16 cases of fracture dislocation in the transverse direction and one in the longitudinal direction. The Day type 2 group consisted of one case of fracture dislocation in the transverse direction and 21 cases in the longitudinal direction. In the Day type 3 group, one case involved fracture dislocation in the transverse direction and 16 cases involved the longitudinal direction.

**FIGURE 4:**
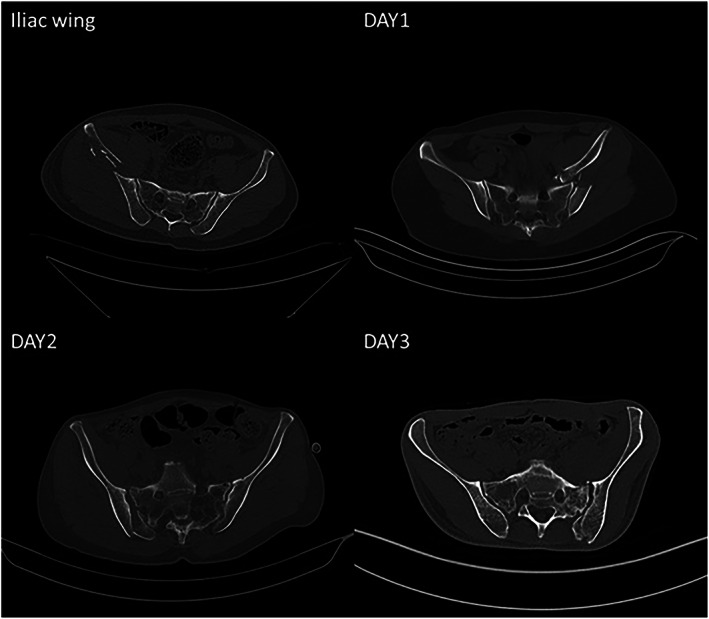
The fracture line and the direction of fracture dislocation in the iliac wing fracture (13/15 patients) and Day type 1 (16/17 patients) groups were transverse; the fracture line and the direction of fracture dislocation in Day type 2 (21/22 patients) and Day type 3 (16/17 patients) groups were longitudinal

The displacement of the fracture was transverse in the iliac wing and Day 1 groups. In the Day types 2 and 3 groups, the internal rotation deformity was in the iliac wing, but the sacroiliac joint dislocation was cephalic and dorsal. In the Day type 3 group, fracture dislocation occurred in the transverse direction in one case and the longitudinal direction in 16 cases. The transverse case and five of the longitudinal cases had minimal displacement. In the other 11 cases, the internal rotation deformity was in the iliac wing and the sacroiliac joint dislocation was both cephalic and dorsal (Figure 4).

#### Displacement Analysis

**FIGURE 5:**
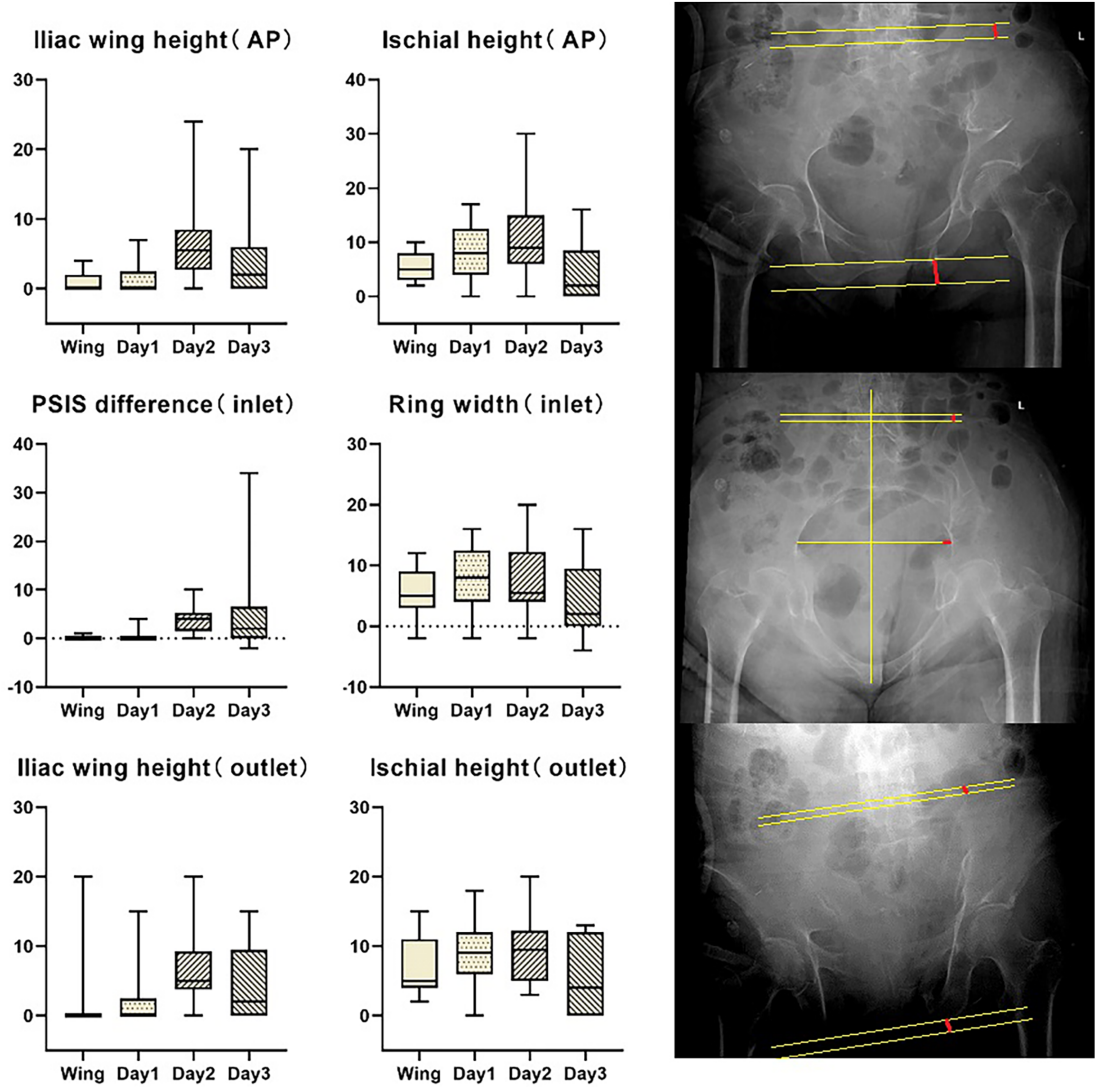
The initial displacement in the four groups was compared and the results were expressed as the median (IQR, interquartile range) (mm). The displacements in the iliac wing fracture and Day 1 groups were practically the same. The displacements in the Day types 2 and 3 groups were also practically the same. All four groups had lateral compression of the pelvic ring and an increase in the ischial height. The differences in the iliac wing height and posterior superior iliac spine in the iliac wing fracture and Day type 1 groups were less than those in the Day type 2 and 3 groups

The initial and postoperative displacements in the four groups were measured in the AP, inlet, and outlet views. The data for the four groups are shown in Table 2. The differences in the initial displacement among the four groups are shown in Figure 5.

**TABLE 2:** Preoperative and postoperative displacement measurements

Displacement Measurements	Iliac wing		Day 1		Day 2		Day 3	
	Preoperative (mm)	Postoperative (mm)	Preoperative (mm)	Postoperative (mm)	Preoperative (mm)	Postoperative (mm)	Preoperative (mm)	Postoperative (mm)
Iliac wing height (AP)	0 (0, 0)	0 (0, 0)	0 (0, 2)	0 (0, 1)	6 (3.25, 8)	2 (1, 3)	2 (0, 5.5)	0 (0, 0.25)
Sacral height (AP)	0 (0, 0)	0 (0, 0)	0 (0, 0)	0 (0, 0)	0 (0, 0)	0 (0, 0)	0 (0, 0)	0 (0, 0)
Ischial height (AP)	5 (4, 7.5)	2 (1, 2)	8 (4, 12)	3 (2, 6)	6 (6.25, 15)	3 (1, 4)	2 (0, 5.5)	0 (0, 1.25)
PSIS difference (inlet)	0 (0, 0)	0 (0, 0)	0 (0, 0)	0 (0, 0)	4 (2, 5)	1 (0, 4)	0 (0, 3.75)	0 (0, 2.25)
Sacral width (inlet)	0 (0, 0)	0 (0, 0)	0 (0, 0)	0 (0, 0)	0 (0, 3)	0 (0, 1)	1 (0, 2)	0 (0, 1.25)
Ring width (inlet)	5 (3, 8.75)	1 (0, 2)	8 (4, 12)	1 (1, 2)	6 (4.25, 12)	1 (0, 2)	4 (0, 7.5)	0 (0, 1)
Iliac wing height (outlet)	0 (0, 0)	0 (0, 0)	0 (0, 2)	0 (0, 1)	5 (4, 9)	2 (1, 3)	2 (0, 7.5)	0 (0, 0)
Ischial height (outlet)	5 (3, 9.75)	3 (1.5, 3)	9 (6, 12)	3 (2, 5)	10 (5.25, 11)	3 (2, 5)	4 (0, 8.25)	1 (0, 3)

Abbreviations: AP, anteroposterior; PSIS, posterior superior iliac spine.

According to the preoperative X‐ray, the displacements in the iliac wing fracture group and the Day type 1 group were practically the same: the crescent bone fragments were large and stable enough to support the iliac wing fragment, which moved inward with rotation in the sagittal plane. The displacements in the Day types 2 and 3 groups were practically the same: the crescent bone fragments were too small to support the iliac wing fragment, which was subjected to internal rotation as well as cephalic and dorsal deformity.

### Clinical Outcomes

The patients were followed up for an average of 31.6 months (range, 12–72 months). Three patients died during the follow‐up period, two died of advanced age, and one died of cardiovascular events. The last valid follow‐up was used to determine the outcomes. According to the Matta scoring system, excellent and good reduction was achieved for 95.8% of patients (Table 3). The average Majeed pelvis score was 91.6 (Table 4). Five patients developed post‐surgical lateral femoral cutaneous nerve injury and fully recovered 3 months after INFIX was removed.

**TABLE 3:** Postoperative Matta radiological scoring

Matta score	All patients	Iliac wing	Day type 1	Day type 2	Day type 3
Excellent	51	12	10	15	14
Good	17	3	6	6	2
Fair	3	0	1	1	1

**TABLE 4:** Postoperative Majeed functional scoring

Majeed score	All patients	Iliac wing	Day type 1	Day type 2	Day type 3
Excellent	65	11	16	22	15
Good	2	2	0	0	1
Poor	1	0	0	0	1
Dead	3	2	1	0	0

## Discussion

We observed two types of posterior ring morphology for LC2 fractures on the basis of internal rotation. A near‐anatomic reduction and good clinical outcomes can be achieved with two different closed reduction strategies.

### Closed Reduction of Pelvic Fracture

The results of this study demonstrated the feasibility of achieving a reduction of less than 5 mm using the appropriate closed reduction method. The acceptance of a near‐anatomic reduction using a closed approach *vs* an absolute open anatomic reduction remains controversial for LC2 injuries.^6^ However, in certain studies, good reduction with a residual displacement of <5 mm has been shown to be the only predictor of good functional results.^16^

In this study, the width of the pelvic ring was the easiest parameter to reduce anatomically, similar to other clinical studies.^8^, ^11^ This can be easily achieved by rolling the LC2 Schanz screw or pulling on the supra‐acetabulum Schanz screw in the pelvic reduction frame. Additionally, the structure of the pelvic ring can be easily observed through the inlet view. The ischial height was the most difficult to reduce for several reasons. First, although the ischial height can be easily observed, it is often ignored during the operation. Surgeons often use the conventional AP, inlet, and outlet views, which provide a poor view of the ischial height. Secondly, the anatomical structure of the ischium is difficult to control. The ischium is dorsal to the acetabulum on the longitudinal traction axis. The anterior ligaments of the acetabulum are tight, but the posterior ligaments are loose. Consequently, intraoperative control of the ischial tubercle requires a combination of femoral traction and LC2 long screw pronation, which is complicated and difficult. However, reduction of the sacroiliac joint is the most important factor. The ischial height reduction appeared less likely to affect the prognosis, but further research is needed.

### Shortcomings of Past Research

The Young–Burgess and OTA classification systems are widely used^4^, ^5^; however, they are missing some key information.^17^ Currently, LC2 fractures are classified as OTA B2.2. It is believed that the dislocation of crescent fractures primarily destroys rotational stability, but the stability in the vertical direction is barely affected.^4^, ^5^ However, recent research revealed that stability in the vertical direction may also be affected in crescent fractures, suggesting that the current understanding of crescent fracture dislocation is incomplete.^17^, ^18^ In our clinical work, we found five cases of this type of injury in the Day types 2 and 3 groups. In these groups, the vertical displacement was >10 mm, and the posterior ring was completely unstable during the examination under anesthesia (EUA).^19^

At present, percutaneous minimally invasive fixation is widely used for pelvic fractures with great results.^20^, ^21^ With the continuous advancement and increasing experience in minimally invasive techniques, the impact of minimally invasive pelvic fracture treatment will continue to improve.^22^ Considering the advantages of minimally invasive techniques, minimally invasive reduction for complex pelvic fractures would provide a significant improvement in patient care and a desirable approach for the control of injuries in orthopaedics.

### Strengths and Limitations

As far as we know, this study was the largest cohort of LC2 fracture treatment research, and the minimally invasive technology adopted was also the development direction of pelvic fracture treatment in the future. The limitations of this study include the single‐center retrospective study design and the small sample size for each subtype despite the significant study size. Additionally, the outcomes from closed reduction could not be compared to the open reduction outcomes at our center or even the closed reduction outcomes in other centers. Further research is required at medical centers and with comparative cohorts, and prospective randomized controlled studies with long follow‐up are needed before the standard of practice can be changed.

## Conclusion

Two types of posterior ring morphology for LC2 fractures were observed on the basis of internal rotation. Two different closed reduction strategies could be used to achieve a near‐anatomic reduction. We believe that surgeons will find this closed reduction approach helpful for the treatment of LC2 injuries while learning about the different types of displacement for the four LC fracture subtypes.

## Author contributions

Yan Wu performed the data analyses and wrote the manuscript; Xuefeng Zhou helped perform the analysis with constructive discussions; Hua Chen and Peifu Tang contributed to the conception of the study.

## Conflict of Interest

Each author certifies that he or she has no commercial associations (e.g. consultancies, stock ownership, equity interest, patent/licensing arrangements, etc.) that might pose a conflict of interest in connection with the submitted article.

## Ethics Statement

All ICMJE Conflict of Interest Forms for authors and clinical orthopaedics and related research editors and board members are on file with the publication and can be viewed on request. All authors are in agreement with the manuscript. Each author certifies that his or her institution approved the human protocol for this investigation, that all investigations were conducted in conformity with ethical principles of research, and that informed consent for participation in the study was obtained.
